# Heterodimeric Barnase-Barstar Vaccine Molecules: Influence of One versus Two Targeting Units Specific for Antigen Presenting Cells

**DOI:** 10.1371/journal.pone.0045393

**Published:** 2012-09-18

**Authors:** Heidi Cecilie Larsen Spång, Ranveig Braathen, Bjarne Bogen

**Affiliations:** Centre for Immune Regulation, Institute of Immunology, University of Oslo and Oslo University Hospital, Rikshospitalet, Oslo, Norway; University of Ottawa, Canada

## Abstract

It is known that targeting of antigen to antigen presenting cells (APC) increases immune responses. However, it is unclear if more than one APC-specific targeting unit in the antigenic molecule will increase responses. To address this issue, we have here made heterodimeric vaccine molecules that each express four different fusion subunits. The bacterial ribonuclease barnase and its inhibitor barstar interact with high affinity, and the barnase-barstar complex was therefore used as a dimerization unit. Barnase and barstar were fused N-terminally with single chain fragment variable (scFv)s targeting units specific for either MHC class II molecules on APC or the hapten 5-iodo-4-hydroxy-3-nitrophenylacetyl (NIP). C-terminal antigenic fusions were either the fluorescent protein mCherry or scFv^315^ derived from myeloma protein M315. The heterodimeric vaccine molecules were formed both *in vitro* and *in vivo*. Moreover, the four different fused moieties appeared to fold correctly since they retained their specificity and function. DNA vaccination with MHC class II-targeted vaccine induced higher mCherry-specific IgG1 responses compared to non-targeted control. Since mCherry and MHC class II are in trans in this heterodimer, this suggests that heterodimeric proteins are formed *in vivo* without prior protein purification. Surprisingly, one targeting moiety was sufficient for the increased IgG1 response, and addition of a second targeting moiety did not increase responses. Similar results were found in *in vitro* T cell assays; vaccine molecules with one targeting unit were as potent as those with two. In combination with the easy cloning strategy, the heterodimeric barnase-barstar vaccine molecule could provide a flexible platform for development of novel DNA vaccines with increased potency.

## Introduction

DNA vaccines are attractive due to their ease of construction, low-cost manufacturing and *in situ* antigen production [1]. Three DNA vaccines have been licensed for veterinary use, and there is a number of ongoing human clinical trials [1]. Although DNA vaccines are efficient in rodents and some larger animals, such as the licensed vaccines for dogs [2], [3] and horses [4], immune responses in humans have so far been disappointing [5]. Several strategies have been used to increase efficiency of DNA vaccines, including electroporation [6], [7], [8] and improved vector design [9].

A well-known method to increase the immunogenicity of protein antigens is to chemically [10], [11], [12] or genetically [13], [14], [15], [16] incorporate the antigen into antibodies or antibody fragments that target antigen presenting cells (APC). This principle has been extended to DNA vaccination by constructing DNA plasmids that encode for APC-specific fusion proteins. Thus, cells transfected *in vivo* by DNA vaccination secrete fusion proteins that enhance delivery of antigen to APC, resulting in improved immune responses [17], [18], [19], [20], [21].

DNA-encoded fusion proteins can be monomeric, containing a single targeting moiety and a single antigen [17], [18], [21]. However, dimeric versions containing two targeting units and two antigenic units have also been used [19], [20]. A side-by-side comparison revealed that a dimeric version was more immunogenic than the monomeric version [22]. The increased immunogenicity of the dimer could be due to a number of factors such as strong bivalent binding to APC, increased delivery of antigen to APC, increased crosslinking of the B-cell receptor (BCR), and immunogenicity of the foreign dimerization motif employed [22].

Symmetric homodimers are restricted to expression of two identical N-terminal and two identical C-terminal fusions. For combinatorial targeting of APC and delivery of antigens, it would be desirable to establish asymmetrical heterodimers where a single molecule could express different N- and C-terminal fusions. We have here explored the bacterial barnase-barstar system [23] for this purpose. Barnase (110 aa) is a secreted extracellular ribonuclease produced by *Bacillus amyloliquefaciens* and barstar (89 aa) is its inhibitor [24] with which the host uses to protect itself. Their very strong interaction (K_D_ of ∼10^−14^ M) [25] is comparable to that of biotin and streptavidin (∼10^−15^ M) [26], which makes this module an attractive dimerization motif for the design of heterodimeric vaccine molecules. In addition, the three-dimensional structure of the barnase-barstar complex [27], [28] shows that the N- and C-terminal ends of both proteins are located sufficiently distant from the dimerization surface to accommodate fusions. Deyev et. al elegantly attached scFv fragments via a small hinge region onto barnase and barstar and used scFv-barnase and scFv-barstar as building blocks for multivalent miniantibodies *in vitro* [23]. Barnase has also been expressed with scFv attached both N- and C-terminally at the same time [29]. These fusion proteins have been produced *in vitro* in *E.coli* [23] as well in transgenic tobacco plants [30] and 293HEK cells [31].

Here, we have attached fusion proteins to both the N- and C-terminal ends of both barnase and barstar via short flexible linkers. APC targeting units were placed N-terminally. We used scFv specific for MHC class II molecules [19], and scFv specific for the hapten NIP (non-binding control). The vaccine antigens were attached C-terminally. The antigens utilized were the fluorescent protein mCherry [32], [33] and scFv of a monoclonal Ig produced by B cell plasmacytoma MOPC315 [19]. *In vitro* analysis after co-transfection of the barnase and barstar into 293HEK cells showed secretion of heterodimers with four functional and available fusion partners. DNA vaccination of mice with these fusion-gene pairs resulted in enhanced antigen-specific antibody responses. It is therefore possible to construct heterodimeric vaccine molecule with four flexible and available fusion arms which can be used for DNA vaccination.

## Materials and Methods

### Mice and Cell Lines

BALB/c mice were obtained from Taconic. T-cell receptor (TCR) transgenic BALB/c mice carrying the λ2^315^ Ig L-chain-specific and I-E^d^-restricted 4B2A1 TCR have been described previously [34]. All animal experiments were carried out in strict accordance with the recommendations in the Guide for the Care and Use of Laboratory Animals of the National Institutes of Health. The protocol was approved by the National Committee for Animal Experiments (Oslo, Norway). All vaccinations were performed under Hypnorm/Dormicum anesthesia, and all efforts were made to minimize suffering. HEK 293E cells were purchased from American Type Culture Collection (ATCC). The BALB/c B cell lymphoma A20 (IgG2a, κ) cell line [35], subline A20–1.11, was obtained from Prof. S. Buus (University of Copenhagen, Denmark). The λ2^315^-specific, I-E^d^-restricted CD4^+^ T cell clone 7A10B2 has been described previously [36]. Cells were cultured in RPMI 1640 (Invitrogen) supplemented with 10% heat-inactivated fetal calf serum (FCS, Biochrom AG), 0.1 mM Non-essential amino-acids (Lonza), 1 mM natrium pyruvat (Lonza), 50 µM Monothioglycerol (Sigma) and 40 mg/ml Gensumycin (Sanofi-avensis Norway AS), at 37°C with 5% CO_2_ in humidified air.

#### Construction of the heterodimeric plasmid vectors

A Fv^NIP^Fv^315^ Vaccibody construct [19] cloned into a pIVEX vector (Fredriksen et al., unpublished results), containing a His tag C-terminally and lacking the *Sfi*I site close to the *BamH*I site, was digested with *Sfi*I and *BamH*I. The *Sfi*I-*BamH*I fragment was subcloned back into pLNOH2(Fv^NIP^Fv^315^), resulting in a vector that lacked a *Sfi*I site and had a His tag C-terminally on the antigenic unit. Plasmids containing the barnase and barstar protein genes were kindly provided by Prof. Dr. Andreas Plückthun (University of Zürich, Zürich) and Prof. Dr. Sergey M. Deyev (Russian Academy of Sciences, Moscow) [23]. The genes were amplified by PCR. For barstar we used the primers; 5′barstar: 5′ GGAGGTAGCAGCGGTGGAATGAAAAAAGCAGTCATTAACGG 3′ and 3′barstar: 5′ CAGGCCGCTGAGGCCAGAAAGTATGATGGTGATGTCG 3′ and for barnase; 5′barnase: 5′ GGAGGTAGCAGCGGTGGAGCACAGGTTATCAACACGTTT 3′ and 3′barnase:5′ CAGGCCGCTGAGGCCTCTGATTTTTGTAAAGGTCTGAT 3′. The hinge region of pLNOH2(Fv^NIP^Fv^315^) was amplified by; 5′h1∶5′GGTGAGTCGTACGCTAGCAA 3′ and 3′h1cys:

5′TCCACCGCTGCTACCTCCTGGGCACCGTGGGCATGTGTGAGTTGTGTCACCAAG 3′ and these two PCR products were template for a new PCR with 5′h1 primer and 3′barstar or 3′barnase primer. The finishing PCR products were digested with *Hind*III and *Sfi*I and subcloned into pLNOH2(Fv^NIP^Fv^315^) with only one *Sfi*I restriction site. The barstar and barnase containing plasmids are denoted Bs and Bn, respectively. The MHC class II targeting unit (scFv^I-E^, from mouse 14-4-4S anti-MHC class II mAb specific for the Ia.7 determinant on I-E molecules) were subcloned using *Bsm*I and *Bsi*WI from an I-E^d^-specific Vaccibody construct described previously [19]. For the *in vivo* experiments, the His-tag was removed to exclude the possibility that the His-tag would interfere with the antigenic unit; the C-terminal end of scFv^315^ in pLNOH2(Fv^NIP^Fv^315^) [19] was digested with *Bbv*CI and *Bam*HI and subcloned into pLNOH2(Fv^NIP^BnFv^315^His) and pLNOH2(Fv^NIP^BsFv^315^His). To make constructs with mCherry, mCherry-his-pLNOH2 [37] was digested with *Sfi*I and *Bam*HI and subcloned into pLNOH2(Fv^NIP^BnFv^315^His) and pLNOH2(Fv^NIP^BsFv^315^His). To remove the His-tag from mCherry, mCherry was PCR amplified with primers flanked by *Sfi*I restriction sites (underlined) 5′-TAGGCCTCAGCGGCCTGATGGTGAGCAAGGGCGAGAGA-3′ and 3′-TAGGCCCTGCAGGCCTCATTACTTGTACAGCTCGTCCATG-5′ and cloned into pLNOH2(Fv^I-E^BsFv^315^) and pLNOH2(Fv^NIP^BsFv^315^). All the different DNA constructs are listed in Table 1.

**Table 1:** Plasmid vectors expressing vaccine polypeptides.

Plasmid name^a^	Targetingunit	Dimerizationunit	Antigenicunit	Tag
Fv^NIP^BnFv^315^His	scFv^NIP^	Barnase	scFv^315^	His
Fv^I-E^BnFv^315^His	scFv^I-E^	Barnase	scFv^315^	His
Fv^NIP^BnFv^315^	scFv^NIP^	Barnase	scFv^315^	-
Fv^I-E^BnFv^315^	scFv^I-E^	Barnase	scFv^315^	-
Fv^NIP^BsChHis	scFv^NIP^	Barstar	mCherry	His
Fv^I-E^BsChHis	scFv^I-E^	Barstar	mCherry	His
Fv^NIP^BsCh	scFv^NIP^	Barstar	mCherry	-
Fv^I-E^BsCh	scFv^I-E^	Barstar	mCherry	-

a scFv from mAb αI-E^d^, αNIP and myeloma protein M315 are denoted Fv^I-E^, Fv^NIP^ and Fv^315^, respectively. Barnase, Barstar and mCherry are denoted Bn, Bs and Ch, respectively.

#### Production and purification of vaccine proteins

The pLNOH2 vectors carrying complete vaccine genes expressed from a CMV promoter were transiently transfected into HEK293E cells using lipofectamine 2000 (Invitrogen) according to the manufacturer’s protocol. 1.2 µg DNA for each plasmid were used per well in 24 wells plates. Supernatant harvested from up-scaled transient transfections of HEK293E cells were purified on a DNP (dinitrophenyl)-lysine-Sepharose column (Sigma). After washing with PBS and the hapten analogue 0,05 M N-carbobenzoxy (CBZ)-glycine (Merck), bound heterodimers were eluted with 0.05 M DNP-glycine (pH 7.4, Sigma) and then passed through DOWEX 1×8−100 (Cl) ion–exchange resin (Sigma) to remove the hapten. Proteins were concentrated using 10 kDa cutoff Vivaspin 20 (Sartorius Stedim Biotech Gmbh).

#### SDS-PAGE and Western blot analysis

Purified proteins were analyzed by sodium dodecyl sulphate polyacrylamide gel electrophoresis (SDS-PAGE) using 4–12% Novex Tris-Glycine Gels (Invitrogen). For non-reducing samples 4 µl of 6x sample buffer (SB), containing 12% SDS, 300 mM Tris pH 6.8 and 0.05% bromophenolblue, was added to 20 µl of sample. For reducing samples 2 µl of 1M DTT (Sigma) was added in addition to the SB. Reduced samples were boiled at 95°C for 10 min before they were loaded onto the gel. Following membrane transfer, proteins were detected with biotinylated 6X His tag® (Abcam) followed by horseradish peroxidase-labeled streptavidin (GE Healthcare).

#### Flow Cytometry for analysis of anti I-E^d^-specificity

A20 1.11 cells were seeded out at 3×10^5^ cells/well in a sharp-bottom 96 well plate (Sterilin), and blocked in 30% rat serum and 100 µg/ml 2.4G2 (anti FcγRII/III Ab) for 20 min. Cells were then stained with heterodimers specific for MHC class II, I-E^d^ in 3-fold dilutions starting at 20 µg/ml, incubated for 45 min. Subsequently, cells were stained with biotinylated anti-mCherry mAb clone 2 (2,0 µg/ml) [37] for 25 min. Finally, cells were stained with 2 µg/ml Streptavidin-phycoerythrin (BD Pharmingen) for 10 min. Washes between stains included centrifugation for 5 min at 300 g, 4°C. All incubation steps were done on ice in the dark. The stained cells were analyzed on a FACSCalibur (BD Biosciences, Mountain View, CA) and data were analyzed using CellQuestPro software (BD Biosciences, San Jose, CA). The concentration of purified αNIP/αNIP heterodimer was determined by Nanodrop (Thermo Scientific) and used as standard in the ELISA to normalize the concentration of the other heterodimers. The ELISA used DNP-BSA as coat and biotinylated anti-mCherry as detection antibody.

#### Analysis of mCherry fluorescence

The different constructs were transiently transfected into HEK293E cells as described above. On day 3, pictures were acquired with an original magnification of ×40 (Nikon ECLIPSE Ti-S inverted microscope).

#### *In vitro* T-cell proliferation

T-cell proliferation assays were performed essentially as described [38]. T cells used were either the λ2^315^-specific CD4^+^ Th1 cell line 7A10B2 or polarized λ2^315^-specific Th2 cells from 4B2A1 TCR transgenic BALB/c mice. BALB/c spleen APC were irradiated at 20 Gy, before 5×10^5^ cells/well and 2×10^4^ T cells/well were added to flat-bottom 96 well cell culture cluster plates (Corning Incorporated) together with heterodimers or synthetic (89–107)λ2^315^ peptide, as a positive control, diluted in a 5-fold series in triplicates. The start concentration of heterodimers was normalized by ELISA as described above and verified on Western blot. The start concentration of the positive control was 10 µg/ml. Negative controls were synthetic (107–119) HA peptide (10 µl/ml), NIP-targeted heterodimers, medium and mock. The mock control was medium purified by the same method as heterodimers with a starting volume equal to that of the highest volume of the heterodimers. The plates were incubated at 37°C/5% CO_2_ for 48 hours before 50 µl medium was removed for IFN-γ and IL-4 analysis. Cultures were then pulsed with 1 µCi [^3^H]dThd for 48 hours and then frozen at −20°C. Finally cells were harvested (Tomtec Harvester) onto glass fiber filters and counted using 2450 Microplate Counter (Perkin-Elmer).

#### *In vivo* DNA vaccination and electroporation

Plasmid DNA was purified using Endofree-mega plasmid purification system (Qiagen). Female 6 to 8 week old BALB/c mice were anesthetized with Hypnorm/Dormicum subcutaneously before vaccination. Intradermal injections with 20 µg DNA of each plasmid (Table 2) in 20 µl of 0.9% NaCl were made on each flank, near the base of the tail immediately followed by electroporation using Derma Vax™ (Cyto Pulse Sciences, MD, USA) [39]. Blood samples were taken from the saphenous vein every week during the first two months, and then every second week.

**Table 2:** Heterodimer plasmid pairs used *f*or DNA immunization.

Name of heterodimer	Plasmid pairs used *in vivo* a	Targeting unit	Dimerization unit	Antigenic unit
αMHCII/αMHCII	Fv^I-E^BnFv^315^	scFv^I-E^	Barnase	scFv^315^
	Fv^I-E^BsCh	scFv^I-E^	Barstar	mCherry
αMHCII/αNIP	Fv^I-E^BnFv^315^	scFv^I-E^	Barnase	scFv^315^
	Fv^NIP^BsCh	scFv^NIP^	Barstar	mCherry
αNIP/αNIP	Fv^NIP^BnFv^315^	scFv^NIP^	Barnase	scFv^315^
	Fv^NIP^BsCh	scFv^NIP^	Barstar	mCherry

a scFv from mAb αI-E^d^, αNIP and myeloma protein M315 are denoted Fv^I-E^, Fv^NIP^ and Fv^315^, respectively. Barnase, Barstar and mCherry are denoted Bn, Bs and Ch, respectively.

#### Enzyme-linked immunosorbent assays (ELISAs)

ELISAs were performed in Costar 96 well plates (Corning Incorporated) coated with Abs or other reagents diluted in PBS with a volume of 60 µl/well and incubated overnight at 4°C. Plates were blocked with PBS containing 10% BSA (160 µl/well) and incubated at 25°C for 1 hour. Samples, standards, and secondary reagents were diluted in ELISA buffer (PBS containing 0.2% Tween and 0.1% BSA, 0.02% Na-azide) in a volume of 50 µl/well and incubated for at least 1 hour at 25°C. Serum samples were incubated overnight. All ELISAs were detected using Streptavidin-ALP (GE Healthcare, 1∶3000), and developed by adding 100 µl/well phosphatase substrate (Sigma). The colour reaction was measured on a TECAN Sunrise Microplate reader. Plates were washed three times with PBST between each layer.

- ELISA for *in vitro* analysis of vaccine protein. Plates were coated with DNP-BSA (5 µg/ml), Ab2–1.4 (mouse mAb specific for scFv^315^, 2 µg/ml) [19], anti-mCherry mAb clone 1 (2 µg/ml ) [37] or NIP-BSA (2.5 µg/ml). Supernatants were diluted 2- or 3-fold and bound heterodimers were detected with biotinylated anti-mCherry mAb clone 2 (1 µg/ml) [37], biotinylated Ab2–1.4 (1 µg/ml) [19] or biotinylated 9A8 rat anti mouse Vλ mAb (1 µg/ml, detects scFv^315^ and svFv^NIP^ having a Vλ2 domain and Vλ1 domain, respectively) [19].
- ELISA for detection of IFN-γ and IL-4 in supernatant. To measure IFN-γ and IL-4 in cell supernatants, samples diluted 2-fold were added to plates coated with 2 µg/ml AN-18 (anti-mouse IFN-γ) [40] or IIBII (anti-mouse IL-4) [40]. Bound cytokines were detected with 1 µg/ml XMG1.2-bio (anti-mouse IFN-γ) [40] or anti-mouse IL-4-bio (BD Pharmingen). Standards used were an IFN-γ preparation (starting at 35.4 ng/ml, 4-fold dilution) [40] or recombinant mouse IL-4 (BD Pharmingen, starting at 2 ng/ml, 2-fold dilution).
- ELISA for detection of antigen-specific antibodies in serum from BALB/c mice. Plates were coated with 2 µg/ml myeloma protein M315 (IgA, λ2) [19] or purified mCherry protein (1 µg/ml). Serum samples were added in series with 3-fold dilutions starting at a concentration of 1∶50 and incubated overnight at 4°C, and bound antibodies were detected with 1 µg/ml biotinylated anti-mouse IgG1_[a]_, IgG2a_[a]_ mAbs (BD Pharmingen) or biotinylated anti-mouse κ mAb (187.1 bio). Endpoint titers were determined as the serum dilution that gave an optical density at 405 nm (OD_405_) of at least twice that observed with serum from a NaCl mouse at 1∶50 dilution. Purified anti-mCherry mAb clone 1 (IgG2a, κ) was used as standard in some of the ELISAs.

#### Statistical analysis

To compare antibody responses of targeted versus non-targeted heterodimers, Student’s *t*-test (unpaired) were performed using GraphPad Prism software. Significance was accepted when *P*<0.05.

## Results

### Design of a Heterodimeric Vaccine Molecule

**Figure 1:**
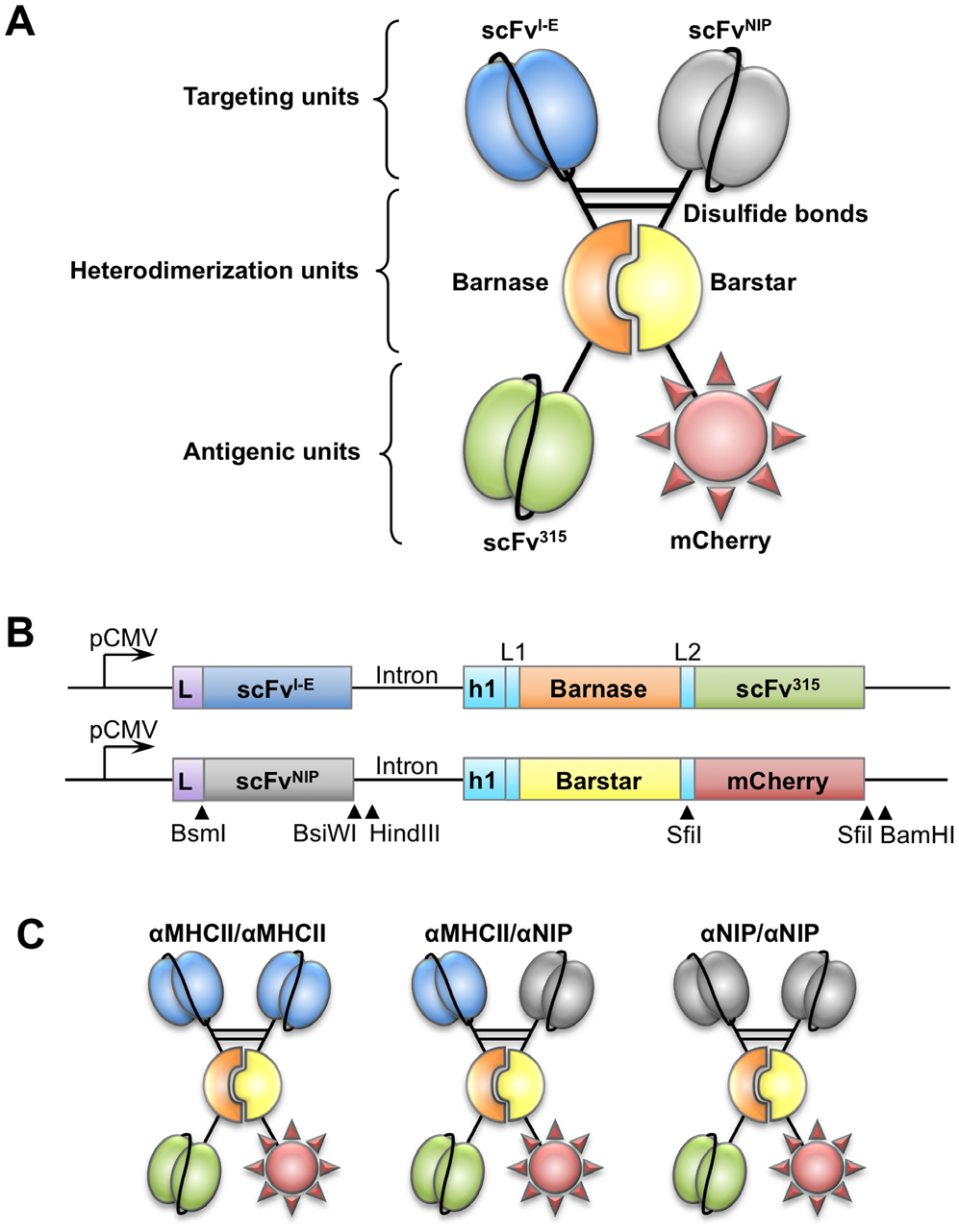
The design of recombinant heterodimeric vaccine molecules. (A) Heterodimerization of polypeptides is obtained by non-covalent interactions between the bacterial ribonuclease barnase and its inhibitor, barstar. N-terminal for both barnase and barstar is a shortened Ig hinge with two cysteines that can form disulfide bonds as depicted in the figure by two black lines. N-terminal of hinges are the targeting units; either scFv specific for MHC class II I-E^d^ (scFv^I-E^, also called αMHCII) or scFv specific for the hapten NIP (scFv^NIP^, also called αNIP), which should not bind any target *in vivo*. C-terminal of both barnase and barstar are the antigenic units, either scFv from mouse myeloma protein M315 (scFv^315^), or the fluorescent protein mCherry. (B) The gene constructs with restriction enzyme sites. The genes are expressed from a CMV promoter (pCMV) and they include the leader sequence (L) found in the pLNOH2 vector. Intronic sequence (Intron), hinge region of human γ3 (h1) and the linkers GGSSGG (L1) GLSGL (L2) are indicated. Two different plasmids, expressing either barnase- or barstar-containing polypeptides, were co-transfected for production of heterodimeric protein molecules. (C) Three different vaccine molecules were made for this study, named after their targeting units; αMHCII/αMHCII that divalently target MHCII^+^ APC, αMHCII/αNIP (monovalently targeted) and αNIP/αNIP (non-targeted). All heterodimeric proteins contain both scFv^315^ and mCherry as antigens within the heterodimeric fusion protein.

We designed vaccine molecules that are heterodimers, with each polypeptide chain consisting of a targeting unit, a heterodimerization unit and an antigenic unit (Figure 1A). The heterodimerization unit consisted of the barnase-barstar module and a shortened immunoglobulin (Ig) hinge region (h1) containing two cysteines. To avoid interference with the formation of incorrect disulfide bonds, a barstar variant lacking two internal cysteines (C40A, C82A) was used [41]. We explored scFv specific for the MHC class II molecule I-E^d^ (scFv^I-E^), as N-terminal targeting unit [19], [20], and scFv specific for the hapten 5-iodo-4-hydroxy-3-nitrophenacetyl (scFv^NIP^) [19] as non-targeted control. Tumor-specific scFv from the BALB/c myeloma protein M315 (scFv^315^) [19] and the fluorescent protein mCherry [32] were added as antigenic units. The gene constructs with restriction enzyme sites are shown in Figure 1B. The targeting, heterodimerization and antigenic units are joined by short linker sequences which should enhance flexibility and correct folding of the separate units. Since we in the present study wanted to address the importance of divalent versus monovalent targeting of APC, we made three distinct vaccine molecules that differ in their targeting units (Figure 1C).

### Characterization of Heterodimeric Vaccine Protein Molecules

**Figure 2:**
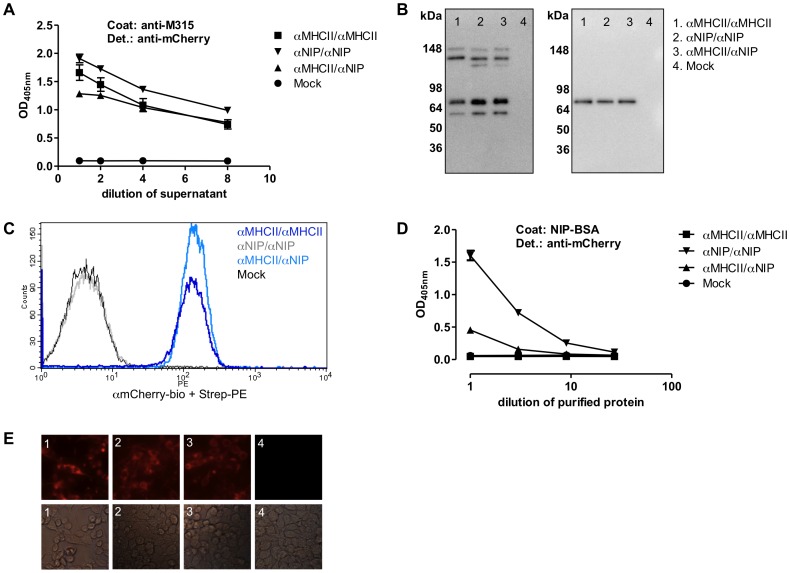
*In vitro* characterization of the heterodimeric vaccine proteins. (A) Supernatants of transiently transfected HEK293 cells were analyzed by ELISA for secretion of heterodimeric vaccine proteins. Ab2.1-4 mAb was used as coat (binds scFv^315^), and bound heterodimers were detected with biotinylated anti-mCherry mAb. (B) Western blot of DNP-purified vaccine proteins probed with biotinylated α6xHis under non-reducing (left) and reducing (right) conditions. (C) MHC class II specificity of proteins measured in flow cytometry. Bound proteins were detected with biotinylated anti-mCherry mAb and Streptavidin-PE. (D) Titration curves of affinity-purified vaccine proteins normalized by Western were analyzed in a NIP-specific ELISA. NIP-BSA was used as coat and bound proteins were detected with biotinylated anti-mCherry mAb. (E) Fluorescence microscopic analysis of transiently transfected HEK293 cells. 1. αMHCII/αMHCII, 2. αNIP/αNIP, 3. αMHCII/αNIP, and 4. Mock. ELISA results are shown as mean ± SD.

To test formation and secretion of heterodimeric vaccine protein molecules, plasmids encoding either barnase- or barstar-fusion proteins were co-transfected into HEK293 cells (Table 2). In initial experiments we observed that mCherry was better secreted when linked with barstar, compared to barnase. For scFv^315^, no such difference was found (data not shown). Thus, as depicted in Figure 1C, we chose to express mCherry linked to barstar and scFv^315^ to barnase. The three vaccine protein molecules, that only varied with respect to targeting units (Figure 1C), were analyzed by ELISA with mAb specific for mCherry and scFv^315^ (Figure 2A). This ELISA detects only heterodimeric vaccine proteins containing both antigens in an assembled two-chain molecule. The results suggest that the *in vitro* heterodimer formation in HEK 293 cells were comparable for the three vaccine proteins. Consistent with this, transient transfections with either the barnase or barstar fusion protein constructs alone failed to induce vaccine proteins detected in the same ELISA (Supplementary figure S1B). The barnase vaccine protein on its own was not secreted from HEK293 cells, but was rescued by the barstar vaccine polypeptide. By contrast, the barstar vaccine protein was secreted even though barnase was not present (Supplementary figure S1C and D).

To further analyze the heterodimers, vaccine proteins in supernatants of transfected cells were purified on a DNP column and analyzed on SDS-PAGE (Figure 2B). The non-reduced blot showed both heterodimers and monomers. Several bands around the expected size of the heterodimers and two bands around the expected size of monomers are believed to be due to disparate glycosylation. Under reducing conditions only a single band was seen corresponding to the expected size of the two monomeric single chains, which happened to be undistinguishable by SDS-PAGE (∼65 kDa). It may be suggested that covalent heterodimerization was only partial, due to incomplete formation of disulfide bonds between cysteines in the hinge regions. However, due to the high-affinity interaction between barnase and barstar, the molecules lacking disulfide bonds might still be noncovalently associated heterodimers that dissociate in SDS.

Both αMHCII/αMHCII and αMHCII/αNIP vaccine molecules bound I-E-expressing cells in flow cytometry (Figure 2C). Similarly αMHCII/αNIP and αNIP/αNIP bound to NIP-BSA in ELISA (Figure 2D) with an expected pattern. These results indicate that the scFv^I-E^ and scFv^NIP^ targeting units folded correctly. Interestingly, we found higher signals for bivalent αNIP/αNIP heterodimers compared monovalent αMHCII/αNIP heterodimers in the NIP-specific ELISA. This suggests that both N-terminal subunits in the αNIP/αNIP heterodimer bound simultaneously, thereby increasing the avidity for the bivalent heterodimeric molecule. However, for the MHC class II-specific targeting unit, we failed to find an influence of bivalency versus monovalency since αMHCII/αMHCII and αMHCII/αNIP bound equally well to I-E^d^ in flow cytometry (Figure 2C). There might be trivial explanations for this discrepancy, e.g., flow cytometry that detects binding of vaccine protein to cells might more poorly distinguish between differences in valency. Although, MHC class II-specific targeting bound equally to I-E^d^ when linked to both barnase and barstar (data not shown), ensuring that both arms of the heterodimers can bind to I-E^d^.

The scFv^315^ and the mCherry antigenic units also appeared to be correctly expressed and folded. The scFv^315^ antigenic unit in the vaccine molecule bound the hapten DNP for which the M315 myeloma protein is specific (data not shown). Further, it was recognized by Ab2.1-4 mAb (anti-Id^315^, Figure 2A) and 9A8 mAb (anti-Vλ, data not shown). Supporting a correct folding of mCherry, 293E cells transiently transfected with the three different heterodimeric plasmid-pairs also emitted red fluorescence (Figure 2E). In addition, mCherry-specific mAb recognized all the heterodimers in ELISAs (Figure 2A and 2D as well as in flow (Figure 2C) and Western analysis (data not shown).

In conclusion, *in vitro* analysis of the three vaccine protein molecules suggests formation of heterodimeric vaccine proteins containing both scFv^315^ and mCherry antigens, and that the four different units are correctly folded and functional.

### MHC Class II- Targeted Heterodimers Induce Antigen-specific Proliferation of CD4^+^ T cells *in vitro*

**Figure 3:**
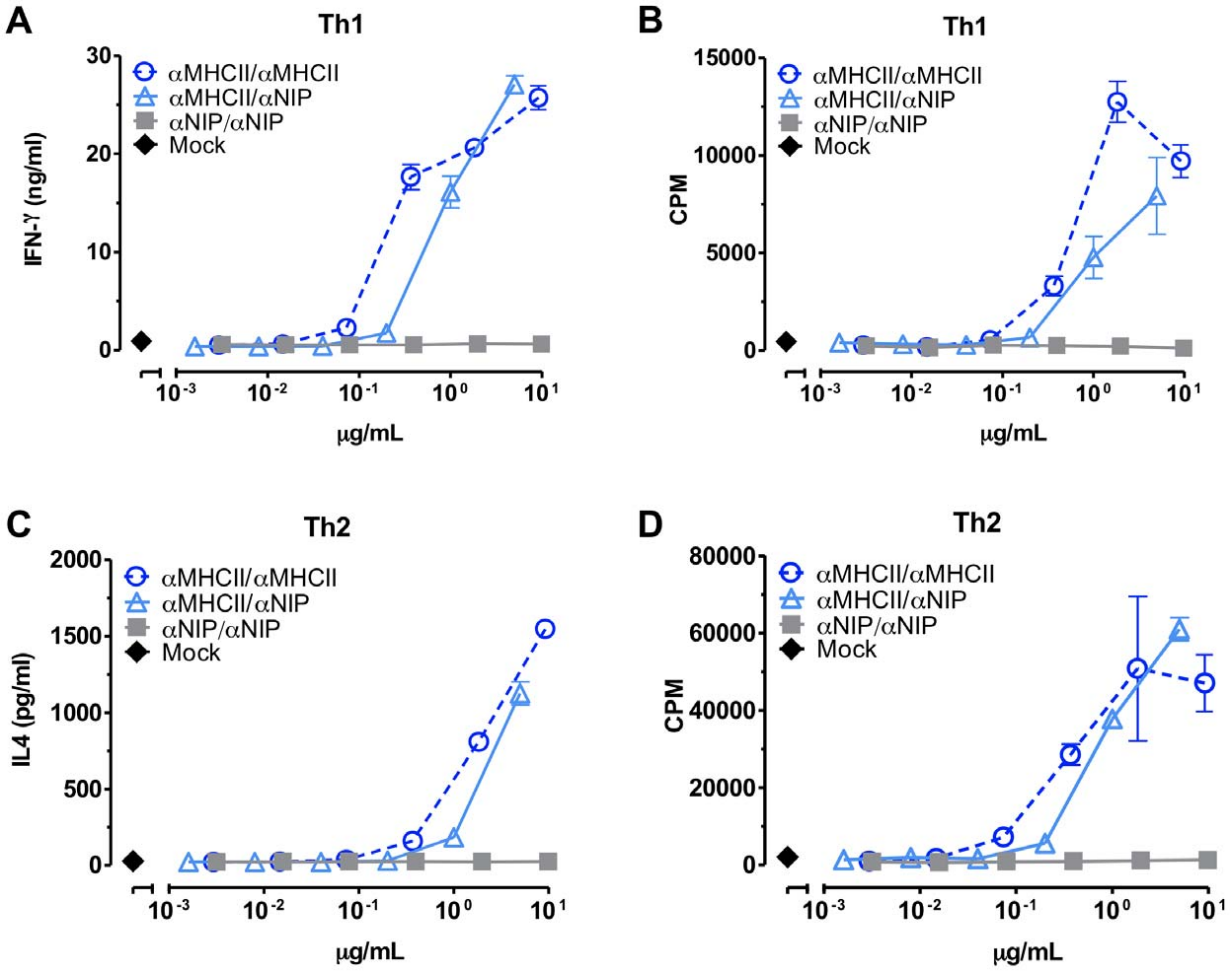
Targeted heterodimers efficiently delivered T-cell epitopes *in vitro*. Titrated amounts of affinity-purified vaccine molecules were added to cultures of irradiated BALB/c spleen APC and CD4^+^ T cells specific for aa 91–101 of the Vλ2^315^ fragment of scFv^315^, presented by MHC class II I-E^d^ molecules. 48 hours later, supernatants were removed for cytokine analysis before addition of [^3^H]dThd. After another 48 hours, T-cell proliferation was measured as incorporation of [^3^H]dThd. (A) IFN-γ secretion by activated cloned Th1 T cells; (B) Proliferation of cloned Th1 T cells; (C) IL-4 production by activated TCR-transgenic Th2 cells; (D) proliferation of TCR-transgenic Th2 cells. The results are shown as mean ± SD.

We analyzed the ability of the three heterodimeric vaccine proteins to induce cytokine secretion and proliferation of T cells recognizing scFv^315^. More specifically, the T cells are restricted for amino acids 91–101 of Vλ2^315^, presented by the MHC class II I-E^d^ molecule. [36], [42], [43]. Irradiated BALB/c splenocytes were used as APC, and T cell responders were either a Th1 cell line (Figure 3A and B) or Th2 cell lines derived from TCR transgenic mice and polarized *in vitro* (Figure 3C and D). Only targeted MHC class II-specific heterodimers induced cytokine secretion and proliferation of antigen-specific CD4^+^ T cells. Importantly, we found no significant difference between bivalent (αMHCII/αMHCII) and monovalent targeting (αMHCII/αNIP, Figure 3), suggesting that only one targeting unit was sufficient for increased targeting to APC and thereby increased CD4^+^ T-cell activation.

### *In vivo* Formation of Heterodimeric Vaccine Molecules Induced Specific Humoral Immune Responses

It is both laborious and time-consuming to produce sufficient amounts of heterodimeric vaccine proteins for immunization of mice. Circumventing this problem, we have previously shown that injection of plasmids encoding either Ig [8] or homodimeric vaccine proteins [19] resulted in transfection of cells that secreted assembled proteins with expected properties. Therefore, to investigate the ability of the heterodimeric vaccine proteins to induce humoral immune responses *in vivo,* we injected pairs of plasmids intradermally (i.d) on each flank of BALB/c mice, followed by electroporation to enhance uptake of DNA into cells [44]. The plasmid combinations used are listed in Table 2. The amounts of scFv^315^- and mCherry-specific antibodies in sera of vaccinated mice were followed for 5 months.

**Figure 4:**
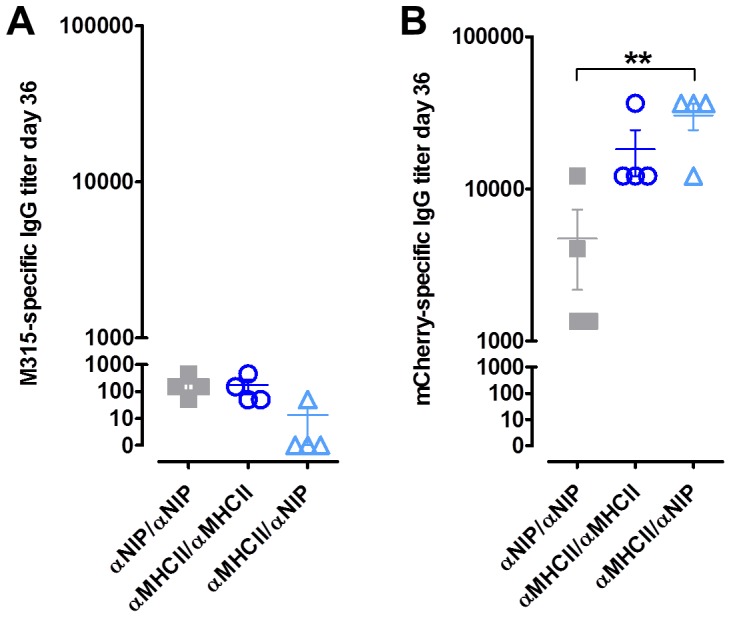
Analysis of antigen-specific antibodies in serum of vaccinated mice. Mice were immunized by intradermal injection of DNA immediately followed by electroporation. The mice were immunized with the indicated DNA plasmid pairs (Table 2). Sera taken 36 days after vaccination were analyzed in ELISA for (A) M315-specific IgG antibodies and (B) mCherry-specific IgG antibodies. ELISA results are shown as mean ± SEM.

All three different heterodimers induced scFv^315^-specific serum antibodies, but there were no significant differences between targeted and non-targeted versions, and the antibody titers were very low, not only at day 36 (Figure 4) but throughout the experiment (data not shown). This observation is consistent with previous findings that a monovalent form of MIP-1α-targeted scFv^315^ hardly induced anti-Id antibodies and that bivalency of scFv^315^ was required. A reason for this might be that scFv^315^ is a weak antigen [45]. Possibly, the low immunogenicity could be even further pronounced when co-expressed with a strong antigen such as mCherry, the latter gaining immunodominance upon vaccination [46].

**Figure 5:**
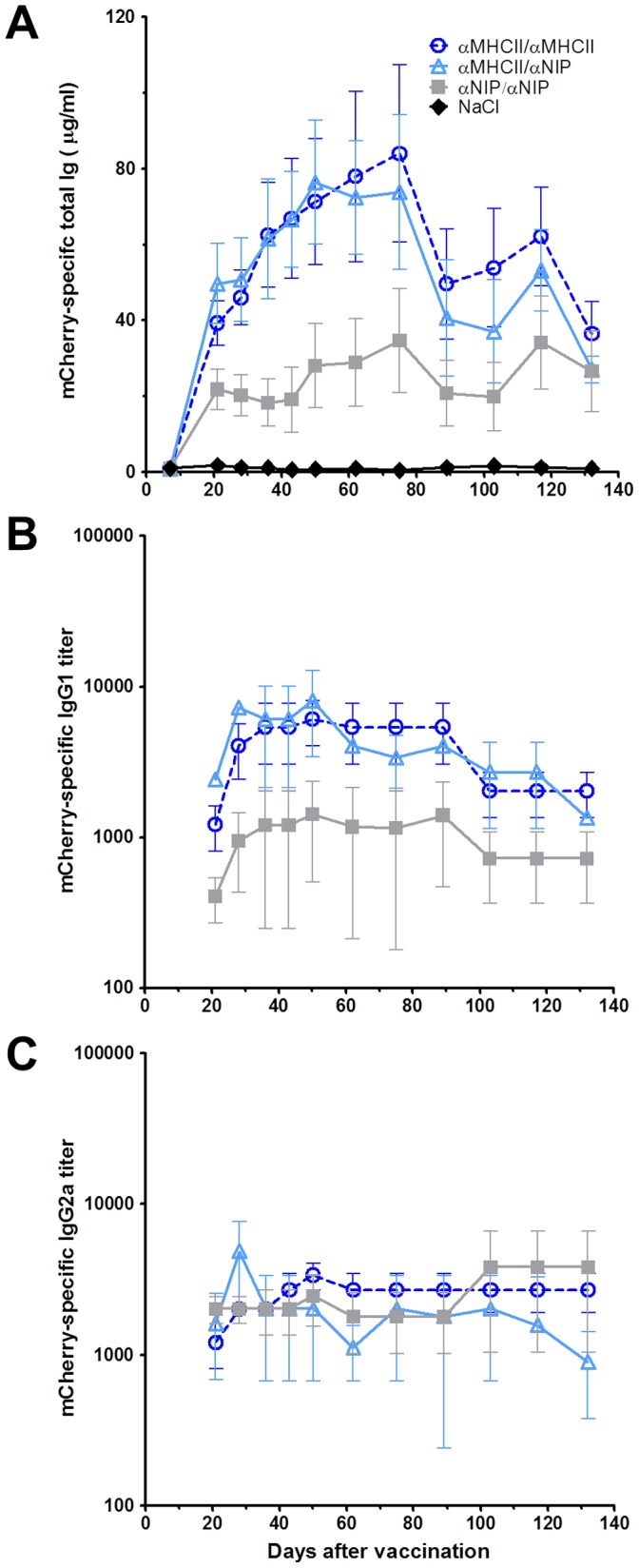
Targeted heterodimers induced high levels of anti-mCherry antibodies. Mice were immunized by intradermal injection of DNA immediately followed by electroporation. Sera obtained at different time-points were analyzed for antigen-specific antibodies by ELISA. Levels of mCherry-specific Ig were measured in sera for 140 days. (A) Total κ^+^Ig anti-mCherry antibodies, (B) IgG1anti-mCherry antibodies, and (C) IgG2a anti-mCherry antibodies. ELISA results are shown as mean ± SEM.

As for induction of total (κ^+^) anti-mCherry antibodies, targeting MHC class II induced high antibody responses at all time points measured, compared to the non-targeted control. Enhancement was particularly pronounced for the first 10 weeks for which the anti-mCherry antibody responses were significantly higher (Student’s t-test). Surprisingly, monovalent and divalent MHC class II-targeted vaccine molecules were equally efficient (Figure 5A).

The same sera were analyzed for BALB/c-specific IgG1 and IgG2a anti-mCherry antibodies (Figure 5B and C). Vaccine molecules targeted against MHC class II, either monovalently or divalently, induced significantly higher titers of IgG1 specific for mCherry compared to the non-targeted molecules. Again, no difference was seen between one or two MHC class II targeting moieties, and the difference to the non-targeted version tapered off with time. For IgG2a antibody responses, MHC class II-targeting had no enhancing effect compared to non-targeted controls. These findings suggest that the combination of MHC class II-targeting, intra-dermal DNA vaccination and maybe also the barnase-barstar motif caused a Th2 response associated with enhanced levels of IgG1 antibodies.

It should be stressed that the increased levels of mCherry-specific antibodies induced by αMHCII/αNIP strongly suggests that heterodimers are formed *in vivo*. This is so because the αMHCII moiety is directly fused via barnase to scFv^315^ while αNIP is fused to mCherry via barstar, i.e., αMHCII and mCherry are in trans in the αMHCII/αNIP heterodimer (Figure 1C). Therefore, because αMHCII/αNIP immunization resulted in enhanced anti-mCherry antibody responses, heterodimers should have formed *in vivo*, otherwise no targeting effect would be seen.

## Discussion

We have designed heterodimeric vaccine proteins that express four fused moieties, both *in vitro* and *in vivo*. As a heterodimerization unit we used the bacterial barnase-barstar module together with parts of the hinge region of human IgG3. Two different targeting units were attached N-terminally, and two different antigenic units C-terminally. When targeted to MHC class II molecules on APC, such heterodimers enhanced CD4^+^ T cell responses *in vitro* as well as induced antibody responses *in vivo*, compared to non-targeted heterodimers.

The *Bacillus amyloliquefaciens* proteins barnase and barstar [24] bind with a very high affinity (K_D_ of ∼10–14 M) [25], comparable to that between biotin and streptavidin. The high affinity between barnase and barstar suggests that they preferentially should form heterodimers. As previously described by Deyev and colleagues [23] barnase is toxic for cells. Consistent with this, we found that singly transfected HEK293E cells did not secrete barnase fusion protein into the supernatant, whereas the barstar fusion protein was secreted. Secretion of barnase fusion protein was rescued upon co-transfection with barstar fusion protein, resulting in assembled heterodimers in supernatants. However, we found that disulfide bonds between h1 Ig hinge exons introduced N-terminally of barnase-barstar module was only formed in a fraction of the molecules, suggesting that not all of the heterodimers were covalently linked.

Heterodimers were also evidently secreted *in vivo* after DNA injection and electroporation. Of particular importance, an enhanced antibody response against mCherry was observed after immunization with the αMHCII/αNIP combination. This strongly suggests that heterodimers are formed *in vivo* because the anti-MHC class II moiety is *in trans* with respect to mCherry in the heterodimer (Figure 1C), and because linkage of the APC-targeting moiety and antigen is known to be required for increasing the immune responses [17].

A previous publication demonstrated that the barnase-barstar module could accommodate fusions with scFv N-terminally of barnase and barstar [23] as well as scFv [29] or a second barnase [23] C-terminal of barnase. Our results confirm and extend this finding since scFv specific for either mouse MHC class II (I-E^d^) or NIP could be expressed N-terminally to barnase and barstar. We did, however, find that heterodimeric constructs with mCherry fused C-terminally to barnase were not secreted into the supernatant and that mCherry did not emit fluorescence (data not shown). However, when mCherry was fused C-terminally to barstar, fluorescent heterodimers were secreted. In contrast to mCherry, scFv^315^ could be fused C-terminally to both barnase and barstar. Thus, there might be restrictions as to what antigens can be fused C-terminally to barnase. It is also possible that C-terminal fusion of mCherry to barnase could be improved by adjusting the linker-sequence between barnase and mCherry.

All four fusion units were functional. This suggests that the heterodimeric vaccine molecule is flexible enough to engage all the four arms simultaneously, however, this remains to be formally demonstrated. Since the cell membrane of an APCs is flexible, it is likely that several different surface molecules may be engaged by a heterodimeric molecule with four different arms. It may be envisaged that either one, two or three targeting units may be combined with either three, two or one antigenic units, respectively. In addition, the targeting and antigenic units may be identical or different. By introducing different targeting units into a single heterodimeric molecule, e.g. MHC class II/TLR/CD40, enhanced signalling and APC function may be obtained. By introducing several different antigens into the heterodimer, the breadth of immune responses may be increased. Such applications would extend the usage of fusion proteins to target APC for enhanced immune responses to infectious or tumor-specific antigen [12], [19], [21].

Here we have tested this idea in a proof-of-principle experiment by generating heterodimers that are either αMHCII/αMHCII (bivalent targeting of APC), αMHCII/αNIP (monovalent targeting of APC) or αNIP/αNIP (non-targeting), but having a fixed mCherry/scFv^315^ antigenic make-up. scFv^I-E^ was chosen because targeting antigen to MHC class II on APC has previously been shown to enhance T-cell and antibody responses *in vitro* and *in vivo* [19], [40]. The increased T-cell responses are most likely due to enhanced uptake of heterodimers by APC, resulting in enhanced presentation of antigenic peptide/MHC molecules. Consistent with this, we show that the scFv^I-E^-containing heterodimers drastically increased T-cell responses, demonstrating that such heterodimers were able to load scFv^315^-derived peptides onto MHC class II molecules. As for B-cell responses, the antigenic units introduced into the heterodimers (mCherry, scFv^315^) are large enough to express conformation-dependent antigenic determinants recognized by BCR of B cells. MHC class II-targeted heterodimers enhanced antibody responses. A likely explanation is that B cells with a BCR that bind the antigenic unit, and that present antigenic peptides on its MHC class II molecules, will receive increased help from effector CD4^+^ T cells that in the first place were stimulated by antigen-primed APC (see above).

The vaccine molecules were designed to test if bivalent targeting of MHC class II on APC was superior to monovalent targeting, as would be expected. Surprisingly, bivalency of anti-MHC class II-targeting was not much superior to monovalency. Thus, while bivalency induced a marginally higher CD4^+^ T cell responses *in vitro*, there was no difference in humoral responses to mCherry. A trivial explanation, could be that only one of the two scFv^I-E^ in the heterodimeric molecule are functional at the same time, since we know that scFv^I-E^ on both arms can bind. Alternatively, bivalency does not add much compared to monovalency.

mCherry was originally included as a marker to follow the production of vaccine molecules *in vivo*, but the results suggest that it also functions as a strong antigen. Both monovalent and bivalent MHC class II-targeting of monovalent mCherry equally enhanced antibody responses compared to non-targeted controls. By contrast, monovalent as well as bivalent targeting of monovalent scFv^315^ to MHC class II, failed to induce any anti-Id antibody responses. This finding is consistent with previous results demonstrating that monovalent MIP-1α-targeting of monovalent scFv^315^ to CCR1,3,5 on APC failed to induce significant anti-Id antibody responses, while bivalent targeting of bivalent scFv^315^ did ([22], Fig 4). Thus, for a strong antigen such as mCherry, monovalent antigen suffices to enhance immune responses upon monovalent targeting. By contrast, for a weak antigen, such as scFv^315^ idiotype, antigens needs to be bivalent even when bivalently targeted to APC.

Our study shows that a heterodimeric vaccine molecule can be delivered by injection of two separate plasmids. This is consistent with the finding that co-injection of plasmids for Ig light- and heavy-chains induce transfected muscle cells to secrete fully assembled Ig [8]. Similarly, injection of a single plasmid induces transfected muscle cells to secrete molecules homodimerized via Ig hinge/CH3 domains [19], [20], [22], [47]. The present study demonstrates that targeted DNA immunization of muscle can be extended to dermal vaccination. In all these cases, electroporation has been employed in order to increase the number of transfected cells which secrete proteins that target surface molecules of APC [8], [48]. The ease of producing vaccines at the DNA level makes this platform a very attractive avenue for constructing and testing new vaccines.

To conclude, we have designed and produced heterodimeric vaccine molecules, with four available and functional arms, that can be delivered as DNA. The technology can in future studies be an important tool box for testing efficacy of various combinations of APC-targeting and various antigens.

## Supporting Information

Figure S1 **Analysis of** ***in vitro*** **secretion of vaccine proteins.** Supernatants of HEK293 cells transiently transfected with pairs of barnase-barstar fusion constructs, as well as with either barnase fusion construct or barstar fusion construct alone, were analyzed by ELISA for secretion of vaccine proteins. (A) Cartoon of the vaccine proteins produced. (B) Ab2.1-4 mAb (specific for scFv^315^) was used as coat and biotinylated anti-mCherry mAb for detection, (C) NIP-BSA was used as coat and Ab2.1-4 mAb for detection, and (D) NIP-BSA as coat and anti-mCherry-bio for detection. The results are shown as mean ± SD.
(TIF)
