# Supplementary figures and images for: Heterodimeric Barnase-Barstar Vaccine Molecules: Influence of One versus Two Targeting Units Specific for Antigen Presenting Cells
Source: PLoS One. 2012 Sep 18;7(9):e45393. doi: 10.1371/journal.pone.0045393 (PMC3445521; doi:10.1371/journal.pone.0045393)

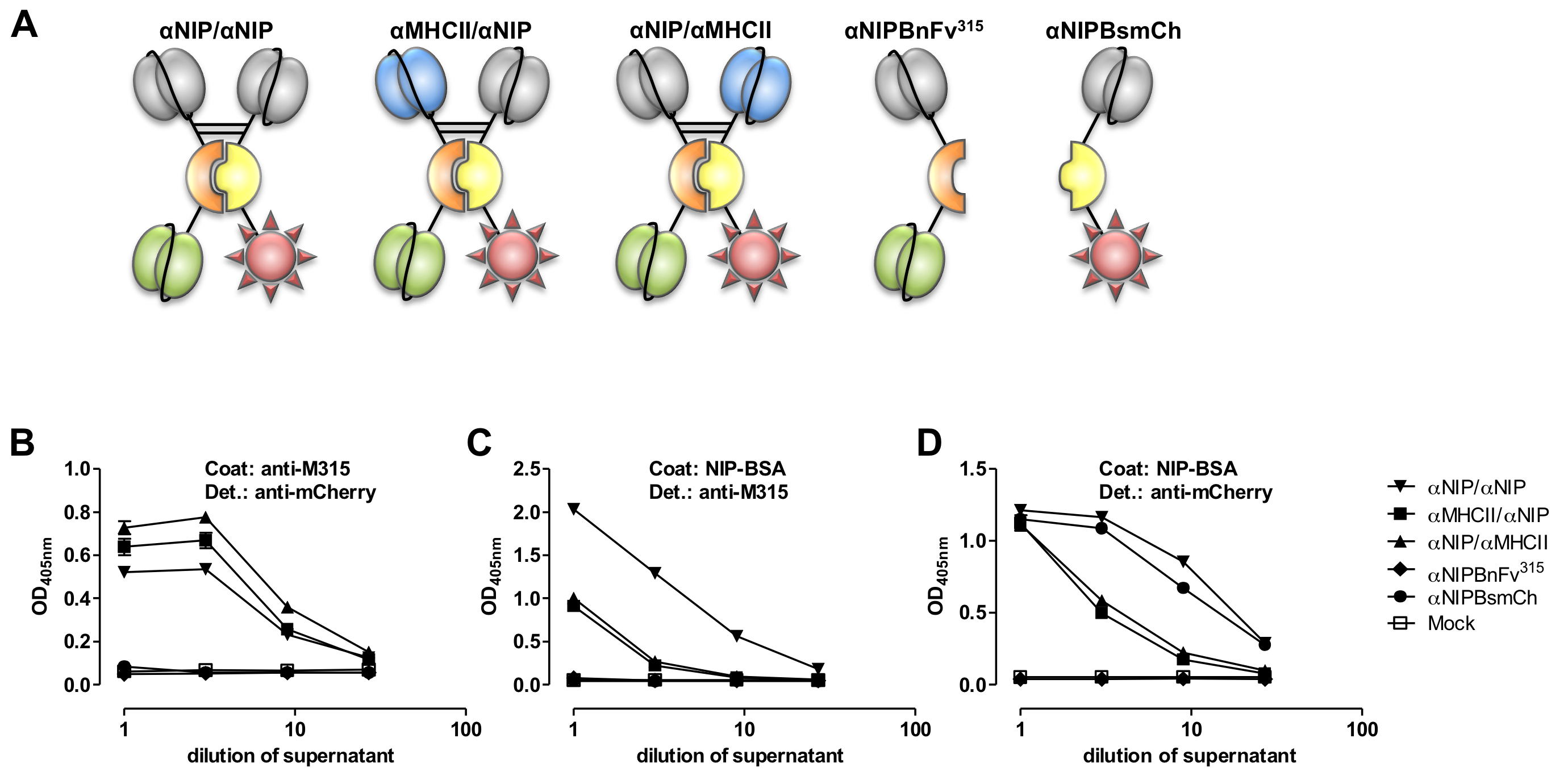

Supplement: Figure S1 — Analysis of in vitro secretion of vaccine proteins. Supernatants of HEK293 cells transiently transfected with pairs of barnase-barstar fusion constructs, as well as with either barnase fusion construct or barstar fusion construct alone, were analyzed by ELISA for secretion of vaccine proteins. (A) Cartoon of the vaccine proteins produced. (B) Ab2.1-4 mAb (specific for scFv315) was used as coat and biotinylated anti-mCherry mAb for detection, (C) NIP-BSA was used as coat and Ab2.1-4 mAb for detection, and (D) NIP-BSA as coat and anti-mCherry-bio for detection. The results are shown as mean ± SD. (TIF) [file pone.0045393.s001.tif]
